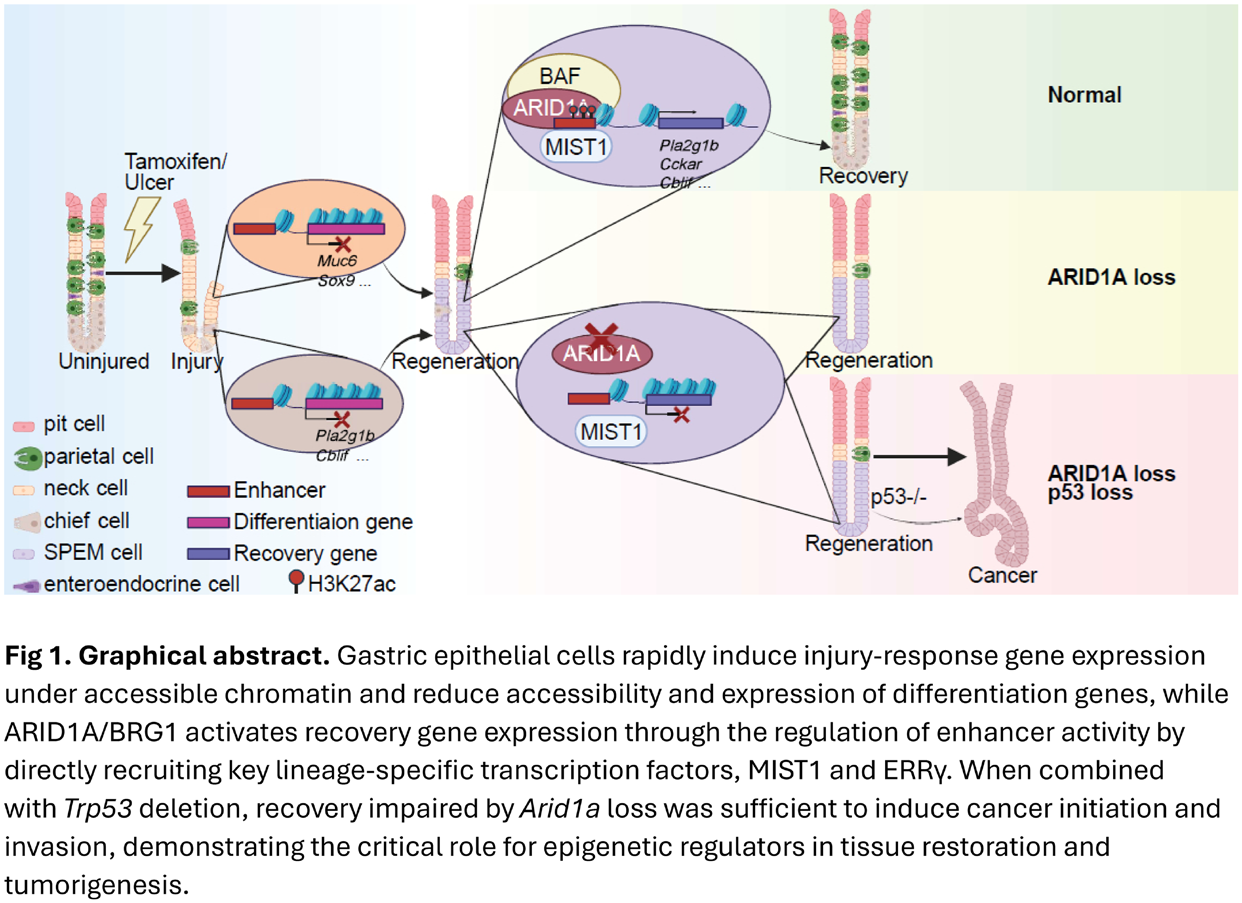# A16 BAF COMPLEX RECRUITS LINEAGE-SPECIFIC TRANSCRIPTION FACTORS TO TERMINATE GASTRIC REGENERATION AND PREVENT CANCER

**DOI:** 10.1093/jcag/gwaf042.016

**Published:** 2026-02-13

**Authors:** A Loe, A Ialongo, Y Qin, A Afkhami, S Hakim, M Saito, B Guan, H Guo, H Rhee, T Kim

**Affiliations:** Developmental and Stem Cell Biology, SickKids Research Institute, Toronto, ON, Canada; University of Toronto Mississauga, Mississauga, ON, Canada; Harvard University, Cambridge, MA; Developmental and Stem Cell Biology, SickKids Research Institute, Toronto, ON, Canada; Providence St Joseph Health, Renton, WA; Fukushima Kenritsu Ika Daigaku Igakubu Daigakuin Igaku Senko, Fukushima, Fukushima Prefecture, Japan; Shandong University Cheeloo College of Medicine, Jinan, Shandong, China; Shandong University Cheeloo College of Medicine, Jinan, Shandong, China; University of Toronto Mississauga, Mississauga, ON, Canada; Developmental and Stem Cell Biology, SickKids Research Institute, Toronto, ON, Canada

## Abstract

**Background:**

The stomach is one of the organs most frequently and directly exposed to environmental insults. It is constantly challenged by physical and chemical stressors from digestion and the acidic lumen, as well as by other environmental factors. The gastric epithelium, therefore, possesses remarkable regenerative capacity and plasticity, enabling both stem cells and specialized cells to respond quickly to injuries and re-establish a functional epithelium. Although abnormal activation of regeneration is known to be associated with cancer, the mechanisms underlying tissue restoration remain unclear.

Gastric cancer (GC) is one of the deadliest and most common cancers in the world, ranking fifth in incidence and mortality. Interestingly, mutations in chromatin modifiers are common in GC. Of note, the core subunit of the BRG1/BRM-Associated Factor (BAF) chromatin remodeling complex, *AT-rich interaction domain 1A* (*ARID1A*), is the second most frequently mutated gene in GC. While the critical roles of Arid1a in GC progression have been demonstrated, its role in gastric regeneration and tissue recovery is unknown.

**Aims:**

We hypothesize that *ARID1A* drives gastric recovery, whereby its loss promote injury-associated tumorigenesis. We aim to define the changes in gene expression and chromatin landscape after gastric injury. We also aim to determine the roles of Arid1a, and its mechanism, in gastric regeneration and recovery.

**Methods:**

We used single-cell gene expression and chromatin analysis to define cell state dynamics during regeneration and recovery. To study the function of *Arid1a* in gastric regeneration and recovery *in vivo*, we analyzed mice with stomach-specific knockout of *Arid1a* using two gastric injury models. We then performed ChIP-seq to study the roles of transcription factors during Arid1a-mediated gastric recovery.

**Results:**

We found that the BAF complex was activated during gastric recovery. Strikingly, deletion of *Arid1a* impaired recovery across multiple injury models, resulting in a persistent regenerative state. Integrative analyses combining single-cell multiome and ChIP-seq demonstrated that the BAF complex recruits lineage-specific transcription factors, MIST1 and ERRγ, to regulate enhancers of recovery genes. Furthermore, deletion of *Trp53* in the unresolved regenerative state caused by *Arid1a* loss is sufficient to drive cancer development and invasion.

**Conclusions:**

We revealed BAF complex as a critical epigenetic mechanism bridging gastric regeneration, recovery and cancer. Notably, the aberration of the BAF complex abolished tissue recovery, leading to an incomplete regenerative state vulnerable to carcinogenesis.

**Funding Agencies:**

CIHROGS, NSERC, GFCC